# Zircon U-Pb geochronology of crystal tuff on Lingshan Island and its geological implications for magmatism, stratigraphic age and geological events

**DOI:** 10.1038/s41598-018-30060-1

**Published:** 2018-08-24

**Authors:** Jindong Gao, Qiao Feng, Xiaoli Zhang, Lifa Zhou, Zunsheng Jiao, Yu Qin

**Affiliations:** 1 State Key Laboratory of Continental Dynamics, Department of Geology, Northwest University, Xi’an, Shaanxi 710069 China; 2 Qingdao National Laboratory for Marine Science and Technology, College of Earth Science and Engineering, Shandong University of Science and Technology, Qingdao, 266590 China; 3 Shaanxi Provincial Institute of Energy Resources & Chemical Engineering, Xi’an, Shaanxi 710069 China; 4 School of Energy Resources, University of Wyoming, Laramie, Wyoming 82017 USA

## Abstract

Due to the unique location in the Ludong region, geochronological study of this area is essential for the understanding of the Cretaceous tectonic evolution of Eastern China. Sedimentary sequences interbedded with tuff layers unconformably overlay metamorphic rocks in the Sulu Orogen. This research presents a more reliable geochronological dataset of a tuff layer on Lingshan Island in Qingdao. A total of 103 valid age values from 216 zircon grains were obtained in three fresh tuff samples. Approximately 87% of these zircon ages are dated as the Early Cretaceous, and their peak ages shift from the Aptian stage to the Albian stage. The spatial-temporal relationship between the tuff and the Mesozoic igneous rocks of Eastern China indicate the impact of the Pacific Plate subduction beneath the Asian continent. Six Albian single detrital zircons have a weighted average age of 103.8 ± 1.4 Ma, with the youngest age (103.4 ± 1.4 Ma) constraining the maximum depositional age of the tuff layer. The age sequence of four sections on Lingshan Island is defined in this study: sections A and B belong to the Laiyang Group, and sections C and D are considered the Qingshan Group and were deposited in the Late Cretaceous. Two pre-Cretaceous zircon age peaks were also observed. These age peaks coincide with the magmatic and metamorphic ages preserved in the Sulu Orogen; thus, the Sulu Orogen is the provenance of the sedimentary rocks on Lingshan Island.

## Introduction

Convergent plate margins are the sites of most intense geological processes, such as magmatism, metamorphism, crust-mantle interactions, and related tectonic activity. Systematic geological, geochemical, and chronological analyses of the igneous rock groups exposed on the margins have provided important clues about the conditions required for the generation of magmas and the geodynamic evolution of an area^1–3^. Terrigenous clastic rocks, pyroclastic rocks, and volcanic lava overlay the ultrahigh-pressure (UHP) and high-pressure metamorphic rock and Yanshan granite in the Sulu Orogen^4,5^. Zhang *et al*. (2013) suggested that the lower strata on Lingshan Island should be the Lingshandao Formation (Fm.) because of the difference in lithology from the Laiyang Group^6^. Lu *et al*. (2011) and Feng *et al*. (2018) proposed that the lower part of the Lingshandao Fm. is a marine lithostratigraphic unit of the Jurassic to Cretaceous, rather than a part of the lacustrine Laiyang Group^7,8^. Li *et al*. (2017) confirmed the sedimentary strata lacustrine facies with the discovery of fish and conchostracan fossils and suggested that it should be the Fajiaying Formation of the Laiyang Group in the Jiaolai Basin^9^. Because the affiliation of the strata is controversial, further geochronology research is needed.

Surprisingly, a tuff layer almost 20.5 metres thick is interbedded in the Mesozoic strata on the Island and is referred to as rhyolite^7^. Based on the weighted mean of eleven concordant ages of 123.9 ± 1.6 Ma, Wang *et al*. (2014) suggested that this tuff was deposited in the Late Aptian^10^. This research presents new and more accurate tuff zircon geochronological data to further constrain the depositional age, and the spatial-temporal correlation between the strata exposed on Lingshan Island and the Mesozoic strata in the Jiaolai Basin. The new tuff zircon geochronological data also allow for mining the records of the pre and syn-collision evolution.

## Geological Setting

### The Sulu Orogen

**Figure 1:**
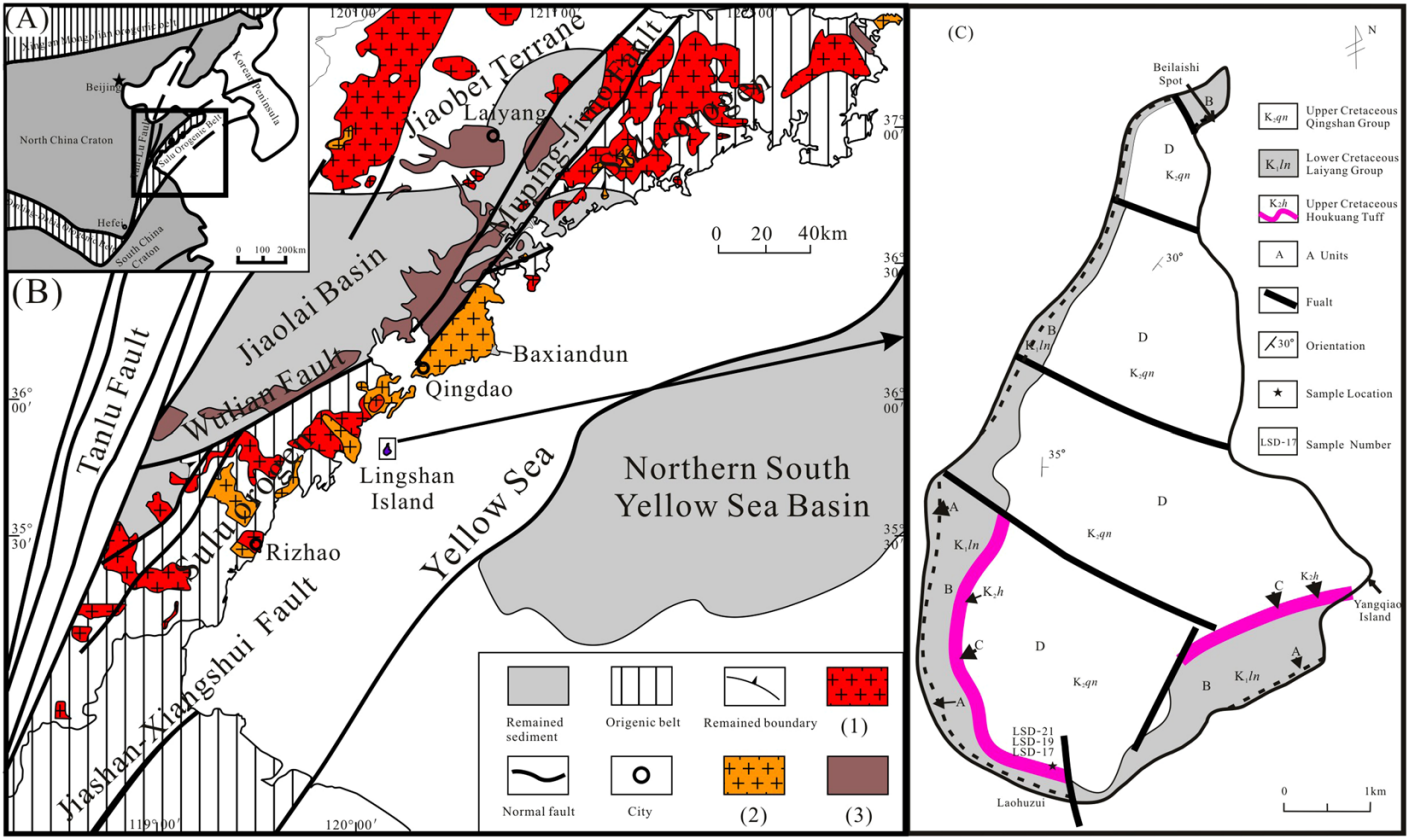
Simplified maps showing. (**A**) The location and tectonic setting of the Ludong area, China (modified after Suo *et al*. and Li *et al*.^87,88^). (**B**) The position of Lingshan Island and geological setting (modified after Suo *et al*. and Li *et al*.^87,88^). (**C**) The geological map of Lingshan Island showing sample locations (modified after Wang *et al*.^10^). And the legends in (**B**) are (1) Linglong granite; (2) Laoshan granite; (3) volcanic rocks.

The Sulu Orogen is the eastern extension of the Qinling–Dabie Orogen (truncated by the Tancheng-Lujiang Fault (TLF)) and developed as the result of the continental collision between the North China Craton (NCC) and the Yangtze Craton (YC) in the Early Triassic^11–13^ (Fig. 1A). The UHP metamorphic rocks are predominantly granitic orthogneiss and enclosed eclogite and record the Neoproterozoic protolith ages and the metamorphism of Early Triassic^14,15^. The Sulu Orogen is not only one of the best-preserved and largest exposed ultrahigh-pressure metamorphic units in the world but also one of the areas that has suffered the most intense degree of magmatic activity during post-collisional exhumation^16,17^. In addition, the prevalent Mesozoic igneous rocks can be divided into 160 to 140 Ma granites represented by Linglong granite and 130–110 Ma granites represented by Laoshan granite (Fig. 1B). Moreover, terrestrial sedimentary rocks are comprised of clastic rocks, pyroclastic rocks, and volcanic lava, which unconformably overlay the UHP to high-pressure metamorphic rocks and Yanshan granite^5^ (Fig. 1B). These strata are truncated by the Wulian-Muping-Jimo Fault and hence have been considered to be outcrops on the edge of the Jiaolai Basin^5^ (Fig. 1B), whereas the lacustrine sedimentary rocks belong to the Laiyang Group, and the pyroclastic rocks in this area belong to the Qingshan Group.

### Geological characteristics of the Jiaolai Basin and Lingshan Island

The Jiaolai Basin is a Mesozoic rift basin in which Early Cretaceous rocks unconformably overlay a Precambrian basement^18^. The stratigraphic sequences comprise the Early Cretaceous Laiyang Group, the Qingshan Group and the Late Cretaceous Wangshi Group, which represents a sequence of lacustrine clastic rock and volcanic rock assemblies^18–20^. Xie *et al*. (2012) and Zhang *et al*. (2004) obtained a maximum sedimentary age of 130 ± 2 Ma for the Laiyang Formation^18,21^. Zhang *et al*. (1996) provided a systematic chronologic analysis of 16 volcanic rocks from each interval of the Qingshan Group and concluded that the age of the Qingshan Group ranges from 125 to 98 Ma^22^. Ling *et al*. (2006) suggested that the age of the Qingshan Group ranges from 106 ± 2 to 98 ± 1 Ma based on a geochronologic study^23^. Qiu *et al*. (2001) concluded that the volcanic eruption age of the Qingshan Group ranges from 103.7 ± 2.8 to 94.0 ± 2.6 Ma^24^. Tang *et al*. (2008) concluded that the age of the Qingshan Group ranges from 118 to 93 Ma^25^. Kuang *et al*. (2012a,b) analysed the volcanic rocks of the Qingshan Group collected from the Qingdao, Jimo, Haiyang, Jiaozhou, Tancheng, Xiguanzhuang regions and concluded that the Qingshan Group ranges from 122 to 99 Ma, according to ^40^Ar/^39^Ar isotopic dating of whole rock^26,27^.

Due to the similarly of the strata on Lingshan Island and in the Jiaolai Basin, the strata sequence of Lingshan Island can be divided into four sections (A to D) from bottom to top based on the characteristics of the rock assemblages (Supplementary Fig. 1). The features of these formations are described as follows.

Section A is mainly of dark grey sandstones interbedded with siltstones and mudstones. Graded-bedding, horizontal bedding (Supplementary Fig. 1a), Bouma sequences and small grooved cross-bedding are exhibited and some structures, such as soft-sediment deformation and convolute bedding, occurr in this area^26^. The top strata overlay the section in a parallel unconformity (Supplementary Fig. 1b).

Yellow, thick-bedded, coarse-grained sandstone (Supplementary Fig. 1c) interbedded with fine sandstone, siltstone and dark mudstones are present in section B. Graded bedding and several convolute beds are exhibited. A Bouma sequence occurs at the top of this section (Supplementary Fig. 1d).

Section C is a thick, grey-white bedded felsic tuff with a maximum thickness of 20.5 m (Supplementary Fig. 1h). The tuff layer has horizontal bedding (Supplementary Fig. 1g) and large horizontal extension characters and conformably overlays Section B (Supplementary Fig. 1e), indicating the continuation of the lacustrine facies of section B^27,28^.

Section D includes conglomerate, pebbly sandstones, sandstones (Supplementary Fig. 1l), grey mudstones, and volcanic rock (Supplementary Fig. 1m), and the upper part of this section mainly comprises volcanic rocks and volcaniclastic rocks (Supplementary Fig. 1o), and multiple mafic dikes. In addition, the uppermost layers incorporate conglomerate and pebbly sandstones (Supplementary Fig. 1n).

## Sample Processing and Analysis

### Petrography of samples

Samples LSD-17, LSD -19 and LSD-21 were collected in the tuff layer at the location of Laohuzui, which is located at the southernmost region of Lingshan Island (Fig. 1C and Supplementary Fig. 1).

Petrographic data indicates that the felsic tuff is mainly volcanic debris, with a small amount of terrestrial debris (Supplementary Fig. 1f,i,j,k). The tuff mainly comprises tephra (>80%) with a minor amount of volcanic breccia (<3%). The tephra is characteristically cuspate-shaped, sinuous and angular. Devitrified debris (<1 mm in diameter) represents a significant part of the rock (40~55%). Small broken quartz and feldspar clasts are interstitial to the matrix and volcanic breccia, and there are small amounts of plagioclase phenocrysts and anhedral grains of haematite in the matrix. Coarse-grained plagioclase phenocrysts are occasionally present and usually exhibit single twinning. Zircon, rutile, and haematite occur as accessory phases in the tuff.

### Analytical methods

Zircons were selected in the Laboratory of the Mineral Geology Research Institute of the Langfang, Hebei Province. The zircon selection, reflected light and cathodoluminescence (CL) imaging, and zircon U-Pb isotope analyses were completed at the State Key Laboratory of Continental Dynamics at Northwest University in Xi’an, China. More detailed experimental procedures and methods can be found in Yuan *et al*.^29^. The obtained isotope ratio data was calculated using GLITTER 4.0 (Macquarie University), and the data was corrected using the external standard of the Harvard zircon 91500. Common Pb corrections were made using the 3D coordinate method following the method of Andersen^30^. Harmonic curve regression analysis was performed using ISOPLOT 3.0^31^. The age data of the zircon was rationally selected. Because zircon contains a large amount of radioactive Pb, ^207^Pb/^206^Pb age data was adopted for ancient zircons with ages of >1000 Ma, and ^206^Pb/^238^Pb age data was adopted for zircons with ages of <1000 Ma^29,32^.

### Analytical results

A total of 103 valid age values out of 216 zircon grains were obtained based on whether their U-Pb analyses were concordant or not. The geochronological data and their Th/U values are listed in Supplementary Tables 1, 2 and 3, and single zircon grain CL images are shown in Supplementary Fig. 2. U-Pb concordia diagrams and their corresponding relative probability plots of U-Pb ages are shown in Supplementary Fig. 3.

The zircons are euhedral and have length/width ratios ranging from one to three. Most zircons are brightly luminescent, with diameters less than 100 μm. Many zircons have retained their original crystal forms, strong oscillatory zoning and Th/U ratios ranging from 0.41 to 6.84, suggesting that they have a magmatic origin^33^. However, zircons 17–57, 21–17 and 21–71 appear homogeneous in CL images and were recorded to possess low Th/U ratios of 0.03, 0.08, and 0.08; these characteristics typically imply a metamorphic origin^34^. Several zircon grains exhibit anhedral dark cores and strong zoning. Supplementary Fig. 3 shows that the test data is concordant or nearly concordant, which suggests a minimal loss of Pb.

The Th/U ratios of the zircons from sample LSD-17 range from 0.48 to 5.22. The U and Th concentrations range from 36.67 × 10^−6^ to 3586.61 × 10^−6^ and from 56.44 × 10^−6^ to 9187.01 × 10^−6^. The ^206^Pb/^238^Pb ages of the samples range from 104.6 ± 2.03 to 758.6 ± 6.79 Ma (Supplementary Fig. 3), and approximately 93.55% of these ages are concentrated between 139.3 and 104.6 Ma, with a peak age of 118.4 Ma.

As for sample LSD-19, more than 98% of the Th/U ratios are greater than 0.4, and the U and Th concentrations range from 41.85 × 10^−6^ to 1960.12 × 10^−6^ and 68.29 × 10^−6^ to 7323.1 × 10^−6^, respectively. Their ^206^Pb/^238^Pb ages range from 103.6 ± 1.39 to 772 ± 9.05 Ma (Supplementary Fig. 3). Approximately 82.1% of these ages are concentrated between 134.6 and 103.6 Ma, with a peak age of 118.2 Ma.

More than 96% of the Th/U ratios are greater than 0.4 for sample LSD-21. In addition, the U and Th concentrations range from 97.09 × 10^−6^ to 1938.96 × 10^−6^ and from 31.47 × 10^−6^ to 5694.25 × 10^−6^, respectively. Their ^206^Pb/^238^Pb ages range from 103.4 ± 1.4 to 769.7 ± 7.44 Ma (Supplementary Fig. 3). Approximately 87.27% of these ages are concentrated between 131.6 to 103.4 Ma, with a peak age of 110.4 Ma.

Overall, more than 80% of the zircon ages are within the Early Cretaceous, with the other recorded ages are older than the Early Cretaceous.

## Discussion

Detrital zircon geochronological research has become a hot topic of global research, as it has been widely used to constrain stratigraphic ages, perform provenance analysis, and provide the inverse analysis of tectonic thermal evolution^35–40^. As products of volcanic activity, tuff layers often exhibit stable distribution and instantaneous deposition. As a result, they are usually regarded as key beds for stratigraphic correlation^41,42^. Moreover, tuff zircon chronology is most useful when obtaining the numerical ages for tephra or transported cryptotephra, although dating the cryptotephra with a high degree of likelihood using stratigraphy and comparisons by matching the inherent compositional features of deposits is common. Hence, this approach is an age equivalent dating method that provides an exceptionally precise volcanic event stratigraphy. Such age transfers are valid because the primary tephra deposits from an eruption essentially have the same short-lived age everywhere they occur and form isochrons quickly after an eruption (normally within one year)^43–47^.

### Interpretation of age data

**Figure 2:**
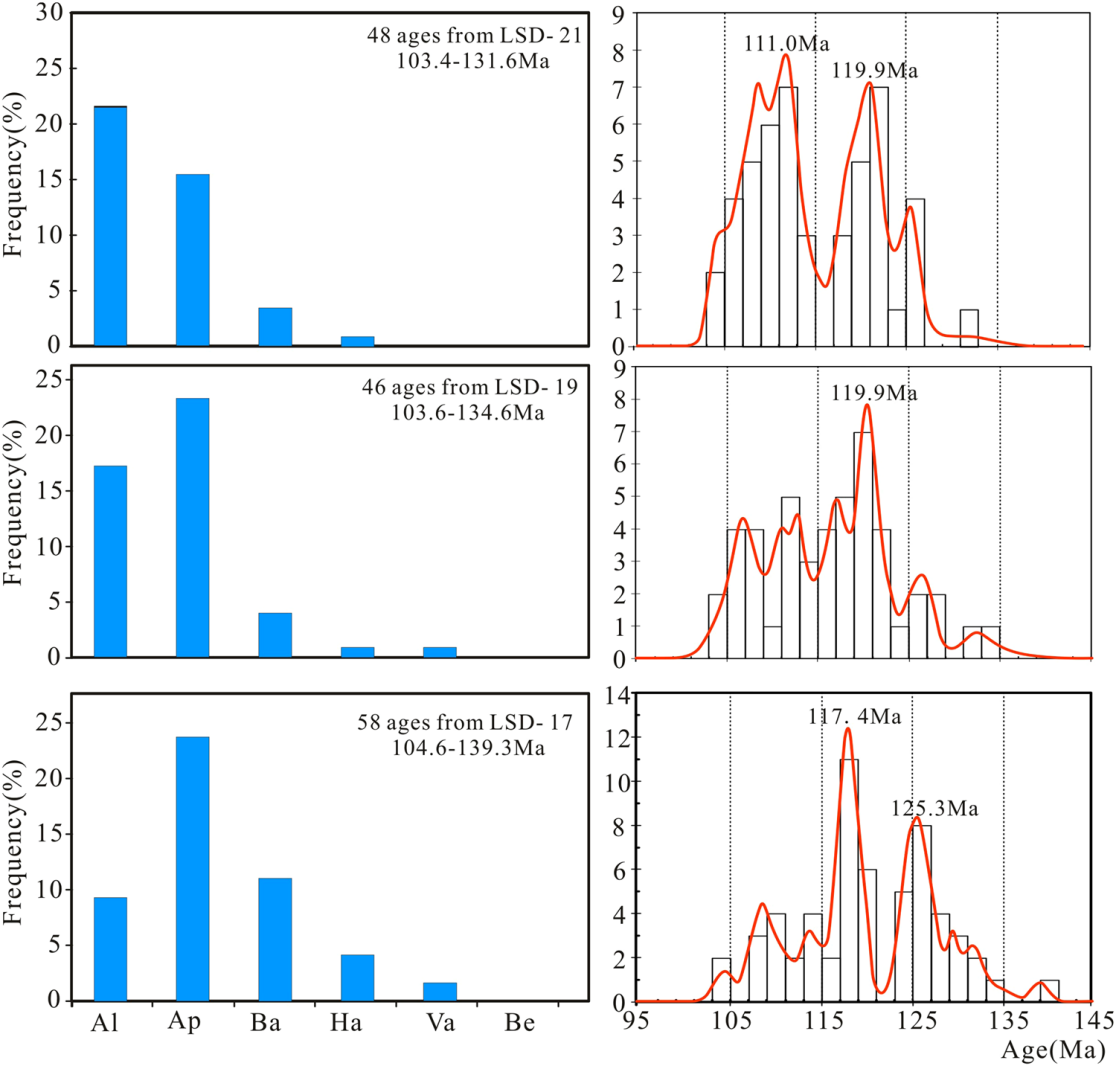
Age-probability plot of the early Cretaceous zircon grains. Notes: Al: Albain; Ap: Aptain; Ba: Barremian; Ha: Hauterivian; Va: Valanginian; Be: Berriasian.

As shown in Fig. 2, the Early Cretaceous zircon age displays the following characteristics: (1) The main peak age shifts from the Aptian stage to the Albian stage; (2) the amount of zircon increases from the Berriasian stage to the Albain stage. The age ranges of the three samples are 104.6 Ma to 139.3 Ma, 103.6 Ma to 134.6 Ma, and 103.4 Ma to 131.6 Ma, respectively, showing that both the maximum and minimum age values decrease as the sample location transitions from the bottom to the top (Fig. 2). These characteristics suggest that these ages are consistent with Smith’s law of stratigraphic superposition^48^, which further supports the accuracy of the obtained age data.

Three peak ages were recorded for the early Cretaceous zircons: ~125.3 Ma, ~119.9 Ma, and ~111.0 Ma (Fig. 2). These age peaks indicate three states of magma condensation and zircon crystallization. Goss *et al*. (2010) obtained a SHRIMP U-Pb age of 115 ± 2 Ma for the Laoshan granite in the Sulu Orogen^49^. That age is the same as the peak age of the Early Cretaceous zircons in our study. Moreover, Early Cretaceous magmatism was widespread throughout Eastern China (Supplementary Table 4) and possesses a NEE directional distribution that diminishes to the west^50–52^. Almost at the same time, the rift basins of China became widely developed^18,53^. All of these facts indicate that the Early Cretaceous magmatism was most likely related to the subduction of the Pacific Plate beneath the Asian continent^18,54^. Therefore, the obtained main age peaks of 125.3 to 111.0 Ma may reflect the background of large scale magmatic activity in Eastern China.

### Age determination of tuff strata

Four methods using detrital zircon to constrain the maximum depositional age were employed in previous studies: (1) the weighted average age of the zircons^1,55–57^; (2) the youngest graphical detrital zircon age^32,58–60^; (3) the youngest detrital zircon age, which is calculated using Isoplot^31,60^; and (4) the youngest single detrital zircon age^61–66^. Because the crystallization of the tuff zircon should be earlier than or contemporaneous with the volcanic eruption and the deposition should be later than the volcanic eruption, the youngest single detrital zircon age is the most reasonable for constraining the maximum depositional age^62,67^.

**Figure 3:**
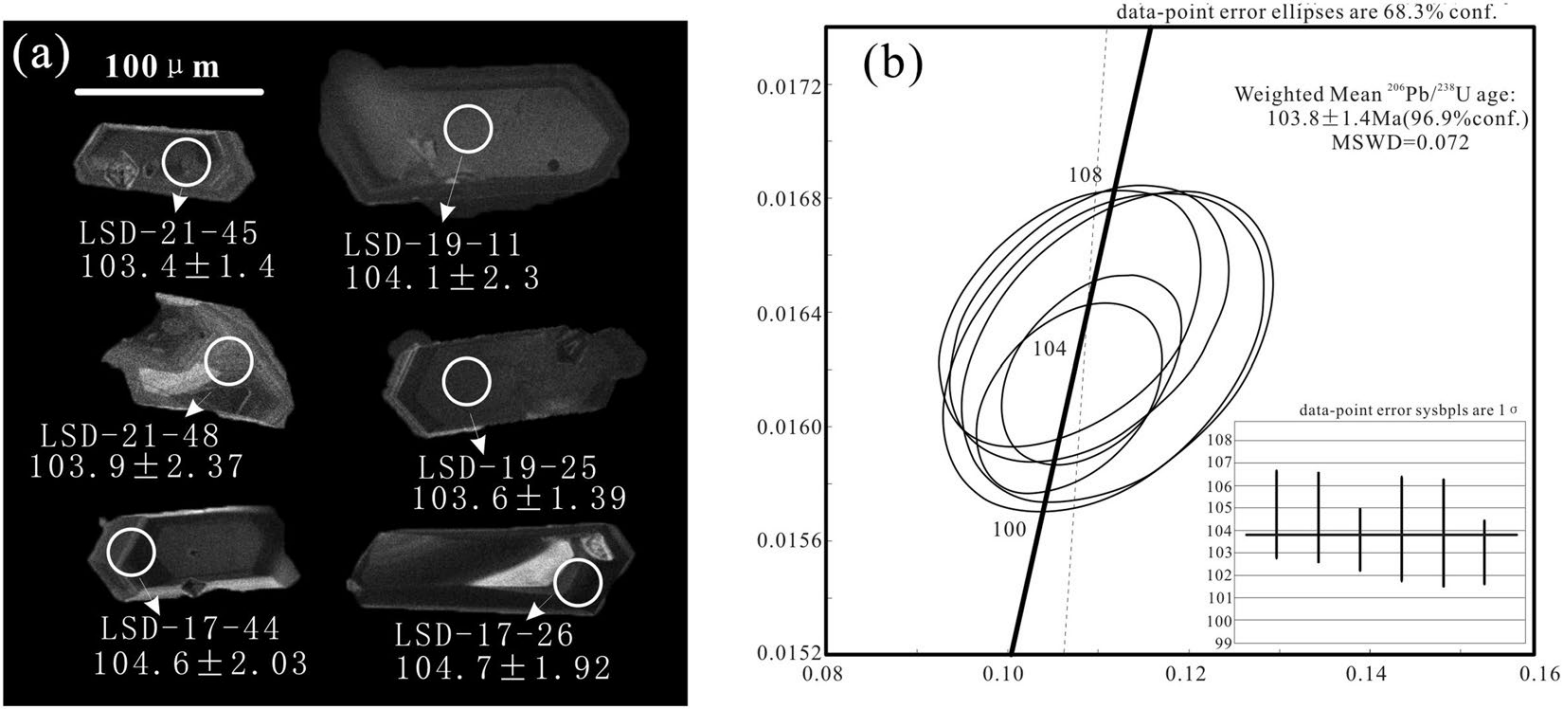
Cathodoluminescence (CL) images of Zircon grains (**a**) and U–Pb concordia diagrams and weighted mean ages in Albian stage (**b**).

Six single detrital zircons were observed with clear oscillatory zoning within the Albian stage. These ages were 103.4 ± 1.4 Ma, 104.1 ± 2.3 Ma, 103.9 ± 2.37 Ma, 103.6 ± 1.39 Ma, 104.6 ± 2.03 Ma, and 104.7 ± 1.92 Ma (Fig. 3a) with a weighted average age of 103.8 ± 1.4 Ma (Fig. 3b). The Th/U ratios were 1.66, 1.05, 1.76, 2.03, 1.96, and 4.17. Ling *et al*. (2006) acquired a weighted average age of the Houkuang Formation of the Qingshan Group in the Jiaolai Basin of 106 Ma^23^. This age is consistent with the obtained age in this research. The youngest single detrital zircon age (103.4 ± 1.4 Ma) constrains the maximum depositional age of the tuff layer, which means that the depositional age of this formation should not be earlier than the Albian period. The age 103.4 ± 1.4 Ma is very close to the boundary age (100.5 Ma) between the Early and Late Cretaceous (ICC, 2015). Moreover, the minimum age of the Qingshan Group is approximately 98 ± 1 Ma^23^. Thus, sections A and B and the Laiyang Group in the Jiaolai Basin were likely deposited in the Early Cretaceous, and sections C and D belong to the Qingshan Group, which was deposited in the Late Cretaceous. This conclusion is different from the hypothesis that the Qingshan Group in the Jiaolai Basin was deposited in the Early Cretaceous and is also different from the conclusion of Wang *et al*. (2014).

### Regional geological events and sediment provenance

**Figure 4:**
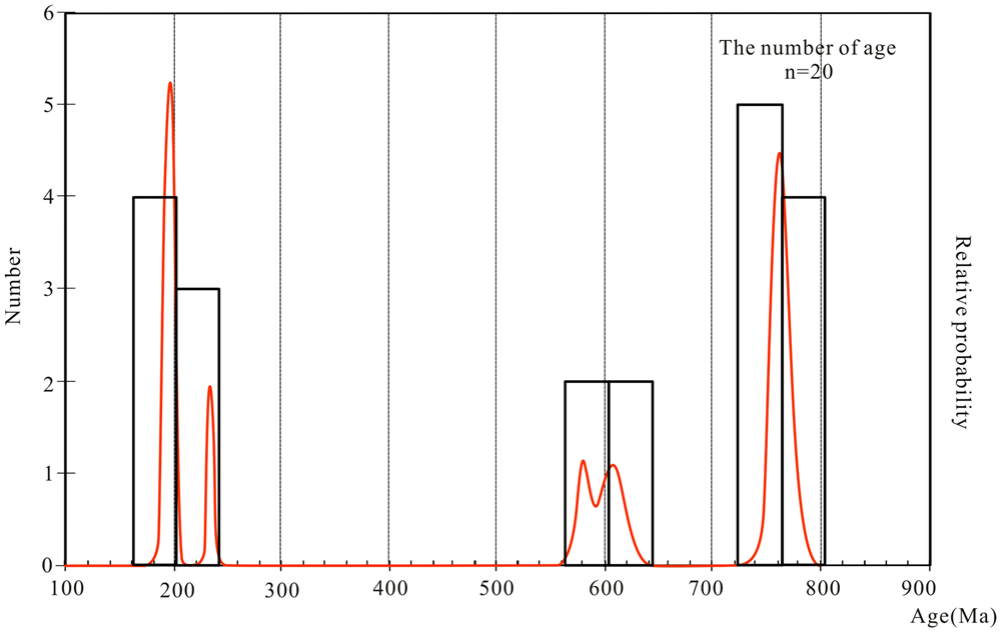
Age-probability plot and age-distribution curve of the pre-Cretaceous zircon grain.

Magmatic activity and metamorphism represent two important records that can be used to determine regional geological events. The geochemical analysis of zircon allows insight into regional volcanic and magmatic processe and also provides an inverse analysis of the tectonic thermal evolution and a sedimentary provenance analysis^68,69^. In this study, we observed two pre-Cretaceous zircon age peaks (Fig. 4), which indicate the prior occurrence of magmatic activity and metamorphism. The ages of the pre-Cretaceous zircons on Lingshan Island coincide with the ages of the pre- and syn-collisional magmatic and metamorphic events^12,70–76^.

#### Precambrian tectonic thermal event (845–582 Ma)

The Precambrian zircons have two secondary peak ages, Early Sinian (772 to 742 Ma) and Late Sinian (609 to 578 Ma) (Fig. 4). An obvious core-rim structure and strong oscillatory zoning indicates that these zircons are of magmatic origin. Granitic orthogneiss with the protolith age of 780 to 570 Ma is widely distributed in the South Sulu Orogen, which indicates that Neoproterozoic magmatic activity was prevalent in the South China Plate^11,12,14,77–79^. The obtained ages are consistent with the age record in the Sulu Orogen. Zheng *et al*. (2013) suggested that Neoproterozoic granitic magma has a characteristic double peak^15^. Geochemical analysis indicated that the magmatic activity was likely related to the breakup of the supercontinent Rodinia^14,80,81^.

#### Triassic-Jurassic tectonic events

There are two secondary peak ages, Triassic and Jurassic. The three individual zircons of Triassic age in this study are 234 ± 2.4 Ma, 206.7 ± 5.1 Ma, and 201.4 ± 1.32 Ma. These zircons are considered to have a metamorphic origin based on their unclear zoning, high degrees of roundness, and low Th/U ratios. Geochronologic values in the Sulu Orogen indicate that the age peak of the high-pressure rocks is 260 to 245 Ma and that their degenerative age is 253 to 210 Ma; the metamorphism peak age of the UHP rocks is 240 to 225 Ma, and their degenerative age is 220 to 200 Ma^76,77,82^. The Triassic detrital zircon obtained in this research is consistent with the metamorphic age, which further supports the age of the continental collision between the North China Plate and South China Plate.

There are five Jurassic zircons with a peak age of 192.1 Ma. These zircons exhibit clear zoning, which reflects their magmatic origin. Lin *et al*. (2000) and Xu *et al*. (2002) reported an Early Jurassic intrusive complex with an age of 191 Ma^83,84^. Zhai *et al*. (2005) suggested that the granite and neutral intrusive rocks formed in the North China Craton at 210 to 180 Ma^85^. These rocks may record the earliest evidence of lithospheric thinning and mantle hydrothermal activity that occurred after the collision of the Sulu Orogen^86^.

## Conclusion

1. The Cretaceous sequence on Lingshan Island is similar to the sedimentary strata assembly in the Jiaolai Basin and is divided into four sections: A, B, C, and D. Section C is a grey tuff layer with a maximum thickness of 20.5 m; the tuff layer possesses lacustrine horizontal facies bedding mainly composed of tephra (>80%) and a minor amount of volcanic breccia (<3%).
2. A total of 103 valid age values out of 216 zircon grains were obtained from three fresh tuff samples. More than 87% of these zircons were dated to the Early Cretaceous. The spatial-temporal relationship between the tuff and Early Cretaceous magmatism of the Ludong area indicate the likelihood of the subduction of the Pacific Plate beneath the Asian continent.
3. Six single detrital zircons within the Albian stage yielded a weighted average age of 103.8 ± 1.4 Ma, with the youngest single detrital zircon age recorded at 103.4 ± 1.4 Ma. The youngest single detrital zircon age is very close to the boundary age (100.5 Ma) between the Early and Late Cretaceous. Thus, sections A and B and the Laiyang Group in the Jiaolai Basin were deposited in the Early Cretaceous, whereas sections C and D belong to the Qingshan Group and were deposited in the Late Cretaceous.
4. Pre-Cretaceous single zircon ages are mainly distributed throughout the Neoproterozoic and Triassic-Jurassic. The Precambrian zircons are likely related to the breakup of the supercontinent Rodinia; the Triassic metamorphic zircons support the geological age of the collision between the North China Plate and the South China Plate, and the Jurassic zircons record the earliest evidence of lithospheric thinning and mantle hydrothermal activity that occurred after the collision of the Sulu orogeny. These facts indicate that the Sulu Orogen is a coeval source provenance of the sedimentary rocks on Lingshan Island.

## Electronic supplementary material

Supplement Table 1

Supplement Table 2

Supplement Table 3

Supplementary Information
