# Supplementary material for: Zircon U-Pb geochronology of crystal tuff on Lingshan Island and its geological implications for magmatism, stratigraphic age and geological events
Source: Sci Rep. 2018 Aug 24;8:12718. doi: 10.1038/s41598-018-30060-1 (PMC6109150; doi:10.1038/s41598-018-30060-1)
Supplement: Supplementary file 4 — Supplementary Information [file 41598_2018_30060_MOESM4_ESM.docx]

**Zircon U-Pb** **geochronology of crystal tuff on Lingshan Island and its geological implications for magmatism, stratigraphic age and geological events**

Jindong Gao ^1, 3^, Qiao Feng ^2★^, Xiaoli Zhang ^1^, Lifa Zhou ^1,3^, Zunsheng Jiao ^3,4^, Yu Qin ^1^

Corresponding author: Qiao Feng E-mail address: 13396251993@163.com

^1^ State Key Laboratory of Continental Dynamics, Department of Geology, Northwest University, Xi’an, Shaanxi 710069, China. ^2^ National Laboratory for Marine Science and Technology; College of Earth Science and Engineering, Shandong University of Science and Technology, Qingdao 266590, China. ^3^ Shaanxi Provincial Institute of Energy Resources & Chemical Engineering, Xi’an, Shaanxi 710069, China. ^4^ School of Energy Resources, University of Wyoming, Laramie, Wyoming 82017, USA


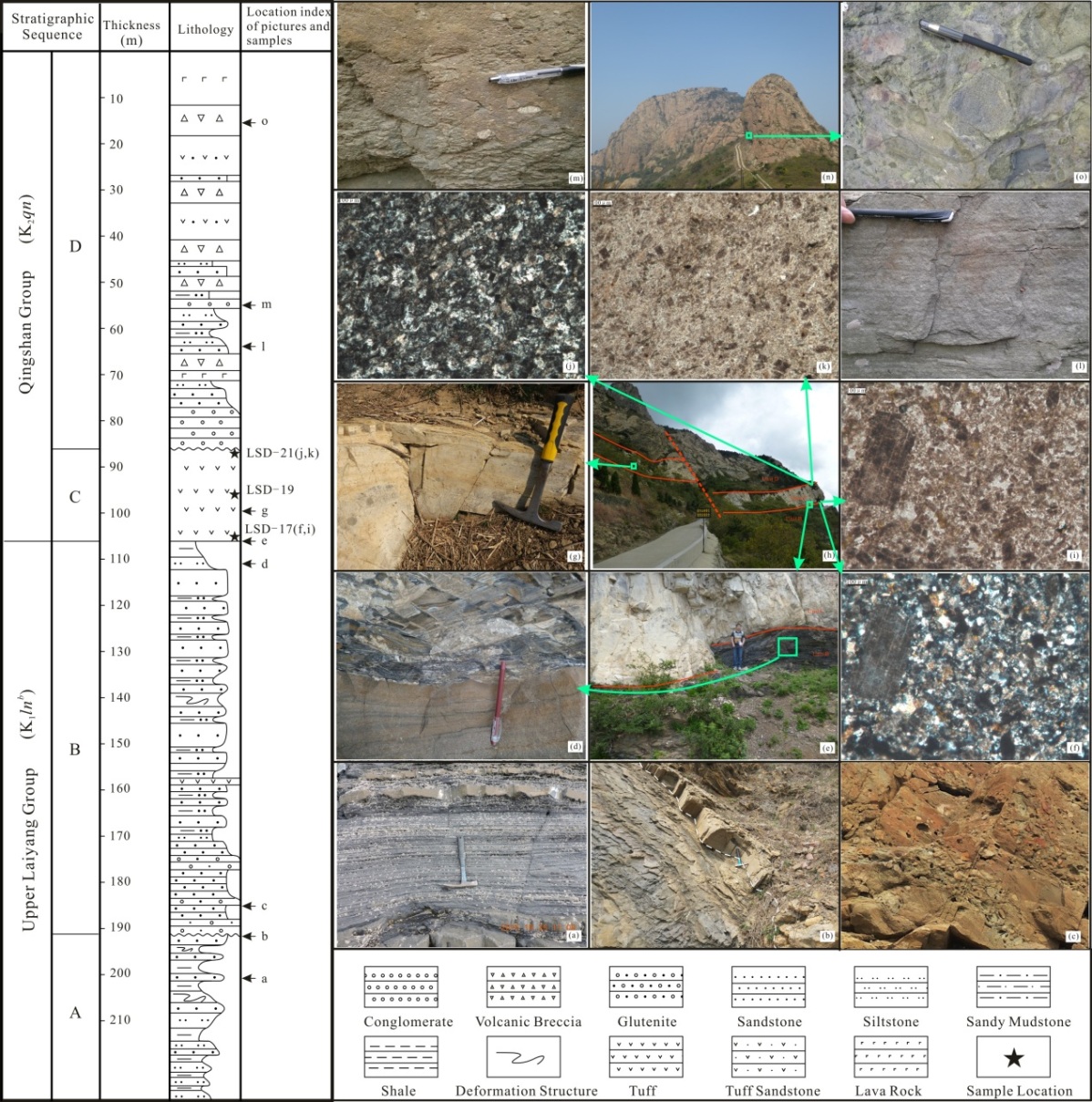


Supplementary Fig. 1 Mesozoic lithostratigraphy on Lingshandao Island with the sampled formation and representative outcrop pictures. Lithostratigraphy modified after Wang et al., (2014) ^[10]^. (a) horizontal bedding; (b) boundary between A and B; (c) sandstone; (d) Bouma sequence; (e) contact relationship between B and C; (g) horizontal bedding in tuff; (h) contact relationship of B, C and D; (m) poorly sorted pebbly sandstones; (n) the top of Lingshan Island; (o) volcaniclastic rock; (f), (i), (j), (k), photomicrographs (polarized light) of samples LSD-19 and LSD-21.


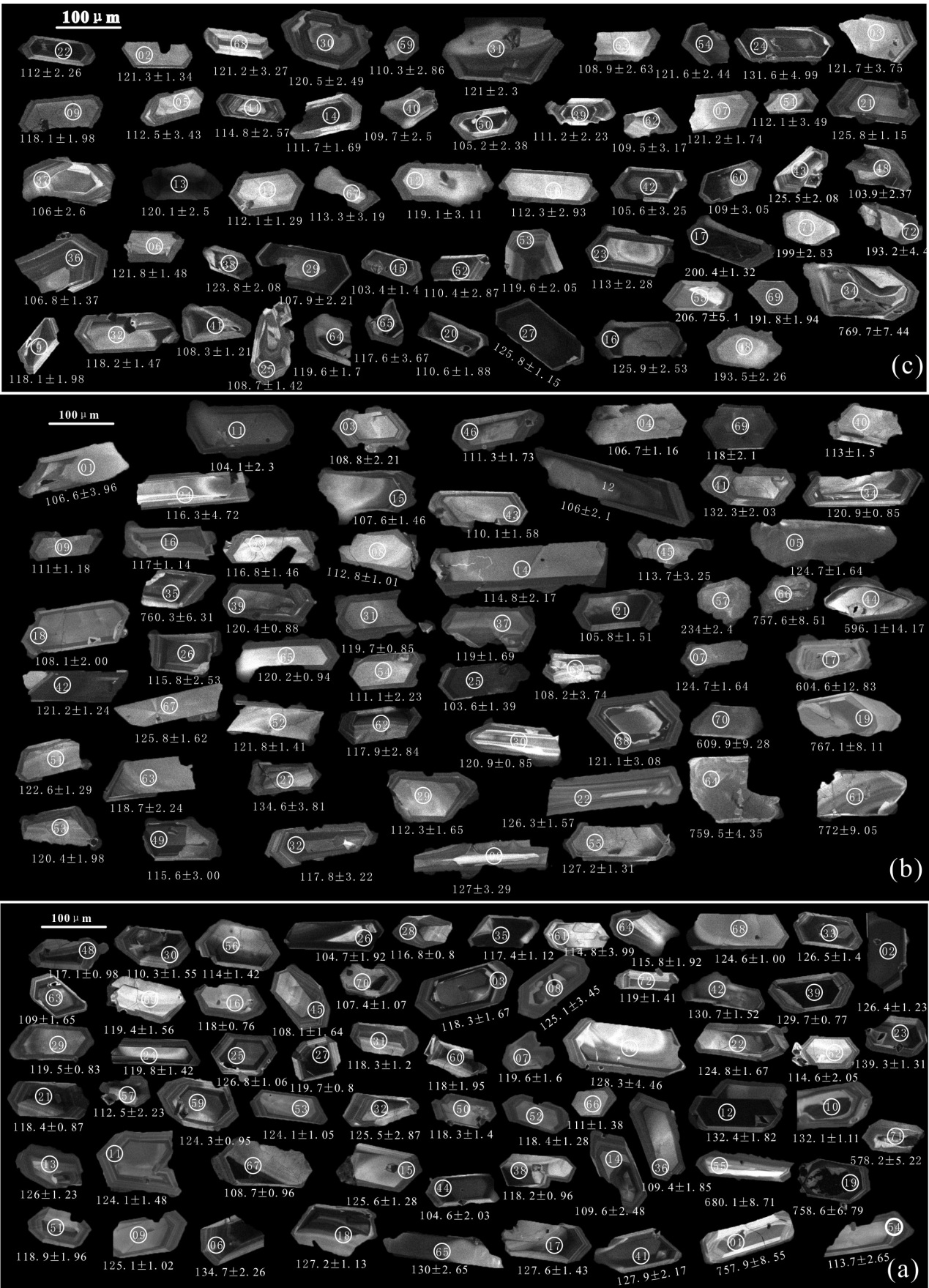


Supplementary Fig. 2 Cathodoluminescence images of zircon from (a) LSD-17, (b) LSD-19, and (c) LSD-21. Circles indicate locations of LA-ICPMS analyses, and numbers represent U–Pb ages. Scale bar is 30 μm for all images.


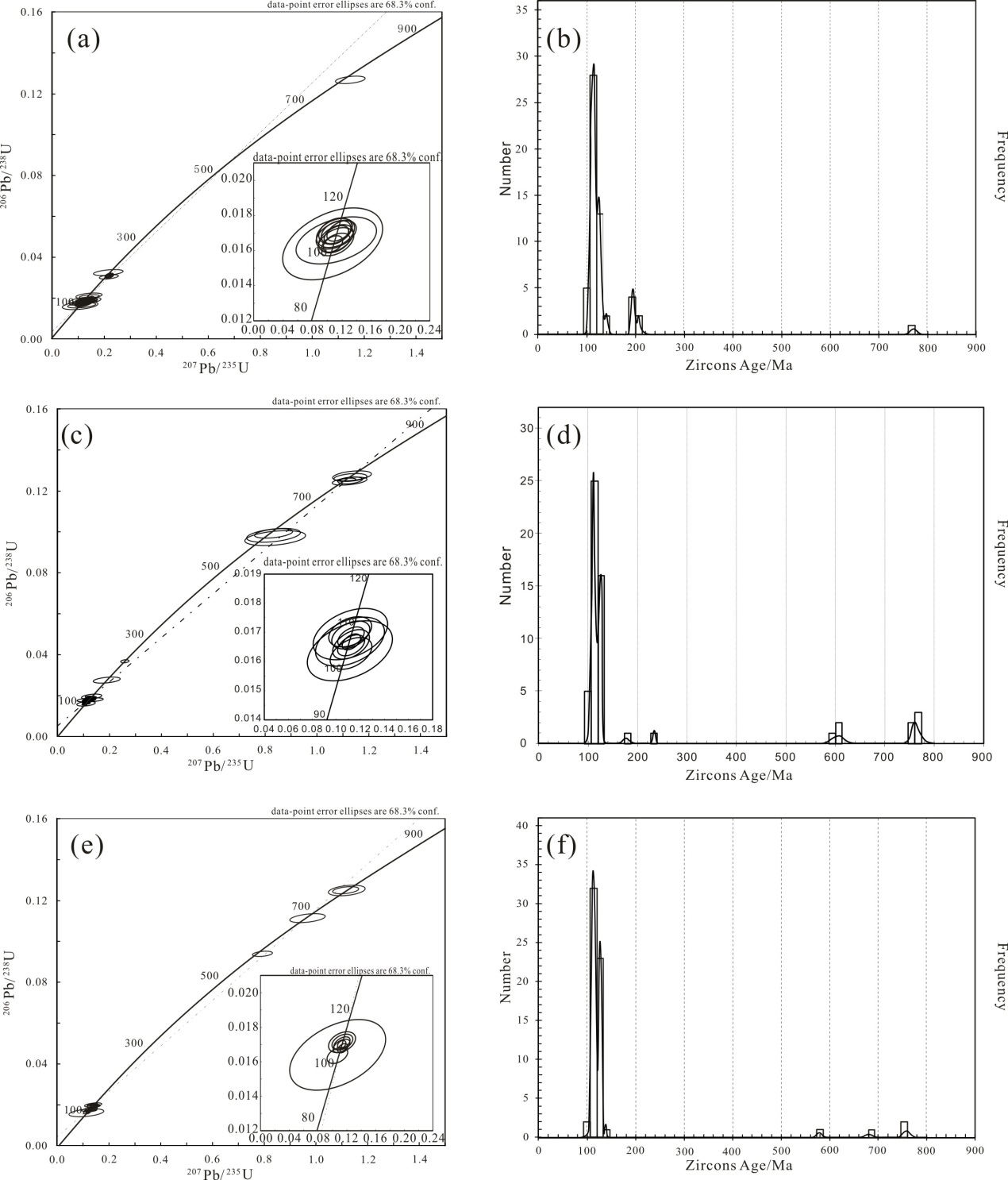


Supplementary Fig. 3 Zircon ^206^Pb/^238^U-^207^Pb/^235^U concordia diagrams (left) and histogram of the zircon grains ages (right) of the tuff in Lingshan island.

Supplementary Table.4 Age statistical table of early Cretaceous magmatic activity in Ludong region ^[92-103]^

| Rock | Lithology | Age（Ma） | Testing technique | Source |
| --- | --- | --- | --- | --- |
| Laoshan mass | Alkali granite, Monzonitic granite | 114±2, 120±4 | SHRIMP | Wang et al., 2009 |
|  | syenogranite,  Alkali granite | 113±0.8, 110.8±0.8 | U-Pb dilution method | Zhao et al., 1998 |
| Weideshan – Zhaohushan –Yashan mass | granodiorite | 117.7±2.9, 113.4±2.5 | SHRIMP | Guan et al., 1998 |
|  | syenogranite | 117.7±2.9 | SHRIMP | Wang et al., 2009 |
|  | syenogranite | 117.7±2.9, 113.4±2.5 | SHRIMP | Qiu et al., 2008 |
|  | Augite diorite | 108±2 | U-Pb dilution method | Guo et al., 2005 |
|  | Gabbro diorite | 113±2 | SHRIMP | Hu et al., 2007 |
| Guojialing mass | granodiorite | 128±2, 126±2, 130±3, 129±3 | SHRIMP | Miao et al., 1997,1998 |
|  | syenogranite | 130~126 | SHRIMP | Guan et al., 1998 |
|  | granodiorite | 125.4±2.2,128.8±2 | LA-ICP-MS  U-Pb | Luo et al., 2014 |
|  | granodiorite | 123~127 | LA-ICP-MS  U-Pb | Yang et al., 2014 |
|  | granodiorite | 129.2±0.6, 129.8±0.6, 130.2±0.7, 129.5±0.7, 129.1±0.8, 129.9±0.6, 129.3±0.8 | ^40^Ar/^39^Ar | Charles et al, 2013 |
|  | granodiorite | 126.2-126.6 | SHRIMP | Li et al., 2017 |

Reference

10. Wang, J., Chang, S. C., Lu, H. B. & Zhang, H. C. Detrital zircon U–Pb age constraints on Cretaceous sedimentary rocks of Lingshan Island and implications for tectonic evolution of Eastern Shandong, North China. *J. Journal of Asian Earth Sciences*. **96**, 27-45 (2014).

92. Wang, S. J. et al. Study on intrusive rocks forming period and stages division in Ludong Area: The evidence from zircons SHRIMP U-Pb age. *J. Jounal of Shandong province land and resources*. **25(12)**, 8-20 (2009).

93. Zhao, G. T., Cao, Q. C., Wang, D. Z. & Li, H. M. Zirconic U-Pb dating on the Laoshan granitions and its significance. *J. Jounal of ocean university of China*. **3**, 382-388 (1997).

94. Guan, K., Luo, Z. K., Miao, L. C. & Huang, J. Z. SHRIMP in zircon chronology for Guojialing suite granite in Jiaodong zhaoye district. *J. Scientia Geologica Sinica*. **33(3)**, 64-74 (1998).

95. Qiu, L. G., Ren, F. L., Cao, Z. X. & Zhang, Y. Q. Late mesozoic magmatic activities and their constrains on geotectonics of Jiaodong region. *J. GEOTECTONICA ET METALLOGENIA*. **32 (1)**, 117-123 (2008).

96. Guo, J. H., Chen, F. K., Zhang, X. M., Siebel, W. & Zhai, M. G. Evolution of syn-to post-collisional magmatism from north Suli UHP belt, eastern China: zircon U-Pb geochronology. *J.* *Acta Petrologica Sinca*. **21(4)**, 1281-1301(2005).

97. Hu, F. F. et al. Pentrogenesis of Gongjia gabbros-diorite in the Kunyushan area, Jiaodong Peninsula: Constraints from petro-geochemistry, zircon U-Pb dating and Hf isotopes. *J. Acta Petrologica Sinica*. **23(2)**, 369-380 (2007).

98. Miao, L. C. et al. Zircon sensetive high resolutionion microprobe (SHRIMPS) study of granitoid intrusions in Zhaoye gold belt of Shangdong provence and its implication. *D. Science in China*. **27(3)**, 207-213 (1997).

99. Miao, L. C., Luo, Z. K., Guan, K. & Kuang, J. Z. The implications of the SHRIMP U-Pb age in zircon to the petrogenesis of the Linglong granite. East Shandong Provence. *J. Acta Petrologica Sinica*. **14(2)**, 198-206 (1998).

100. Luo, X. D., Yang, X. Y., Duan, L. A. & Sun, W. D. Geochemical and geochronogical study of the gold-related Guojialing pluton and Shangzhuang pluton in Jiaobei block. *J. Acta Geological Sinica*. **88(10)**, 1874-1888 (2014).

101. Yang, Q., Santosh, M., Shen, J. & Li, S. Juvenile vs. recycled crust in NE China: Zircon U-Pb geochronology, Hf isotope and an integrated model for Mesozoic gold mineralization in the Jiaodong Peninsula. *J. Gondwana Research*. **25(4)**, 1445-1468 (2014).

102. Charles, N. et al. Timing, duration and role of magmatism in wide rift systems: Insights from the Jiaodong Peninsula (China, East Asia). *J. Gondwana Research*. **24(1)**, 412-428 (2013).

103. Li, H. K. et al. The Mesozoic Magmatic activities Framework in Jiaodong Area: SHRIMP Chronology Recording of Single Particle Zircon. *J. ACTA GEOLOGICA SINICA.* **91 (1)**, 163-179 (2017).
